# Co-designing person-centred quality indicator implementation for primary care in Alberta: a consensus study

**DOI:** 10.1186/s40900-022-00397-z

**Published:** 2022-11-08

**Authors:** Kimberly Manalili, Catherine M. Scott, Brenda Hemmelgarn, Maeve O’Beirne, Allan L. Bailey, Michel K. Haener, Cyrene Banerjee, Sue P. Peters, Mirella Chiodo, Fariba Aghajafari, Maria J. Santana

**Affiliations:** 1 Department of Community Health Sciences, Cumming School of Medicine, 3D10, University of Calgary, 3280 Hospital Drive NW, Calgary, AB T2N 4Z6 Canada; 2 Department of Sociology, University of Calgary, 2500 University Drive NW, Calgary, AB T2N 1N4 Canada; 3 Faculty of Medicine and Dentistry, University of Alberta, 2J2.00 Walter C Mackenzie Health Sciences Centre 8440 112 St. NW, Edmonton, AB T6G 2R7 Canada; 4 Department of Family Medicine, University of Calgary, 2500 University Drive NW, Calgary, AB T2N 1N4 Canada; 5 Department of Family Medicine, University of Alberta, 5-16 University Terrace, 8303 112 St., Edmonton, AB T6G 1K4 Canada; 6 Grande Prairie Primary Care Network, 11745 105 St #104, Grande Prairie, AB T8V 8L1 Canada; 7 Patient and Community Engagement Research Program (PaCER), University of Calgary, 3280 Hospital Drive NW, Calgary, AB T2N 4Z6 Canada; 8 Health Quality Council of Alberta, 210, 811 14 St NW, Calgary, AB T2N 2A4 Canada; 9 Department Paediatrics, Alberta Children’s Hospital, University of Calgary, 28 Oki Drive NW, Calgary, AB T3B 6A8 Canada; 10 Patient Engagement Platform – Alberta Strategy for Patient Oriented Research, University of Calgary, 3280 Hospital Drive NW, Cal Wenzel Precision Health Building, Calgary, AB Canada

**Keywords:** Person-centred care, Quality indicators, Primary care, Implementation, Strategies, Context consensus, Participatory research

## Abstract

**Background:**

We aimed to contribute to developing practical guidance for implementing person-centred quality indicators (PC-QIs) for primary care in Alberta, Canada. As a first step in this process, we conducted stakeholder-guided prioritization of PC-QIs and implementation strategies. Stakeholder engagement is necessary to ensure PC-QI implementation is adapted to the context and local needs.

**Methods:**

We used an adapted nominal group technique (NGT) consensus process. Panelists were presented with 26 PC-QIs, and implementation strategies. Both PC-QIs and strategies were identified from our extensive previous engagement of patients, caregivers, healthcare providers, and quality improvement leaders. The NGT objectives were to: 1. Prioritize PC-QIs and implementation strategies; and 2. Facilitate the participation of diverse primary care stakeholders in Alberta, including patients, healthcare providers, and quality improvement staff. Panelists participated in three rounds of activities. In the first, panelists individually ranked and commented on the PC-QIs and strategies. The summarized results were discussed in the second-round face-to-face group meeting. For the last round, panelists provided their final individual rankings, informed by the group discussion. Finally, we conducted an evaluation of the consensus process from the panelists’ perspectives.

**Results:**

Eleven primary care providers, patient partners, and quality improvement staff from across Alberta participated. The panelists prioritized the following PC-QIs: ‘Patient and caregiver involvement in decisions about their care and treatment’; ‘Trusting relationship with healthcare provider’; ‘Health information technology to support person-centred care’; ‘Co-designing care in partnership with communities’; and ‘Overall experience’. Implementation strategies prioritized included: ‘Develop partnerships’; ‘Obtain quality improvement resources’; ‘Needs assessment (stakeholders are engaged about their needs/priorities for person-centred measurement)’; ‘Align measurement efforts’; and ‘Engage champions’. Our evaluation suggests that panelists felt that the process was valuable for planning the implementation and obtaining feedback, that their input was valued, and that most would continue to collaborate with other stakeholders to implement the PC-QIs.

**Conclusions:**

Our study demonstrates the value of co-design and participatory approaches for engaging stakeholders in adapting PC-QI implementation for the primary care context in Alberta, Canada. Collaboration with stakeholders can promote buy-in for ongoing engagement and ensure implementation will lead to meaningful improvements that matter to patients and providers.

**Supplementary Information:**

The online version contains supplementary material available at 10.1186/s40900-022-00397-z.

## Plain English summary

Person-centred care (PCC) is a model of care where patient needs and preferences are included in decisions about care and treatment. To improve PCC in primary care in Alberta, Canada, we plan to use person-centred quality indicators (PC-QIs). Using PC-QIs involves surveying patients about their care experiences and using this information to make improvements. For example, if 20% of patients do not feel they are getting enough information, the clinic may create a checklist for the providers so information is not missed. We engaged a panel of 11 people, including patients, family doctors, and staff who support quality improvement in clinics across the province to decide together which PC-QIs primary care clinics in Alberta should use. We also asked the panel to decide the most important strategies that would make using the PC-QIs more successful. The panel chose PC-QIs related to: patient and caregiver involvement in decisions about care and treatment, a trusting relationship with the healthcare provider, having health information technology to support PCC, partnering with communities in healthcare, and the patient’s overall experience. The most important strategies were: developing partnerships among people working in primary care in Alberta, discussing their needs and common efforts for improving PCC, engaging “champions,” and securing funding that would be needed. Finally, we asked the panelists to share their experiences with participating in this process. Panelists found the process useful and that their input was valued. Most panelists would also like to continue to work together to put the PC-QIs into practice.

**Supplementary Information:**

The online version contains supplementary material available at 10.1186/s40900-022-00397-z.

## Background

Person-centred care (PCC) is a fundamental approach to primary care [1, 2]. While PCC is integral to delivering high quality primary care, the measurement of PCC is not currently performed in a routine, standardized manner [3–5]. This impacts the capacity of primary care organizations to identify gaps in their provision of PCC and drive the changes needed to enhance the quality of care and improve patient experiences and outcomes [3]. Person-Centred Quality Indicators (PC-QIs), developed by Santana et al. [6] offer an opportunity to address these gaps in measurement and to enhance person-centred primary care practice. The PC-QIs align with the goals of the Patient Medical Home Model [7] to promote PCC and continuous quality improvement (QI), as well ongoing initiatives to incorporate the patient experience into QI [8]. While these quality indicators have been developed, they have not yet been implemented in practice.

Over the past 20 years, there has been considerable progress with respect to the identification and use of implementation strategies in healthcare and other sectors (e.g. education, social programs), which are critical to the promotion of effective implementation, as they aim to mitigate barriers and leverage facilitators to implementation [9, 10]. The methods associated with identifying and applying implementation strategies continue to evolve, including the development of taxonomies for strategies, as well as a variety of systematic approaches [11]. Such systematic approaches include: group model building (engaging stakeholders in a guided approach to identifying and implementing solutions), concept mapping (engaging stakeholders in brainstorming, organizing, and rating strategies, and conjoint analysis (rating-based approach to measure stakeholder preferences, where there are trade-offs with various strategies) [11]. However, questions remain regarding the tailoring of strategies that will reflect a thorough understanding of context [10]. While there is a greater need for systematic approaches to tailor strategies, the integration of more participatory and pragmatic approaches can facilitate stakeholder engagement, particularly among those who are implementing the intervention and are impacted by it [12]. Tailoring strategies also ensures that local (specific implementation setting) contextual considerations and needs are addressed and the most effective strategies are prioritized [10, 13]. Active participation by stakeholders is recognized as an important factor for the success of implementation, and is critical in the planning stages [14]. The engagement of stakeholders is necessary for understanding the context of implementation in primary care, which can be a complex setting. The organization, culture, and structures are highly variable, compared to acute care settings [2, 15, 16].

The literature regarding stakeholder involvement in implementation processes, and particularly with regards to planning for implementation and prioritizing implementation strategies, is limited [12, 17]. Moreover, little is known about the preferences and experiences of stakeholders regarding their participation in planning for implementation [12]. As a participatory and pragmatic research approach that actively engages stakeholders, consensus methods offer an opportunity to collaborate with patients, physicians, and primary care QI staff to seek general agreement on planning for successful PC-QI implementation [18]. A consensus process can help to consider a diversity of perspectives and experiences with PCC measurement (logistics required, capacity, motivations) and how PC-QI implementation may impact them. The collaborative and co-designed nature of the consensus process increases stakeholder ownership over the implementation [19], and thereby increases the likelihood of adoption, implementation, and sustained use, and scale up of the PC-QIs.

The overall aim of our study was to contribute to the development of practical guidance on PC-QI implementation to promote adoption and use in primary care in Alberta, Canada. To work towards this aim, our objectives were to conduct a consensus process for: 1. prioritizing the PC-QIs, and 2. prioritizing evidence and theory-informed implementation strategies previously identified through engagement of stakeholders in PCC measurement across Canada as well as primary care stakeholders in Alberta. In consideration of the limited documentation of the use of consensus processes for prioritizing implementation strategies, our study also included a brief evaluation of the process with the consensus panel members. This study is part of an ongoing program of research to develop and implement PC-QIs for general health system-use in Canada [6], and was approved by the University Health Research Ethics Boards (REB15-2846) at the University of Calgary.

## Methods

### Study design

We used a mixed methods consensus methodology to seek agreement among diverse perspectives represented by Alberta primary care stakeholders [20]. Leech and Ongwuegbuzie’s “Guidelines for conducting and reporting mixed research in the field of counseling and beyond” [21] and Staniszewska et al.’s “Guidance for Reporting Involvement of Patients and the Public” [22] were used to guide the reporting of this study. We drew on the processes associated with a Nominal Group Technique (NGT) approach to obtain consensus with the goal of establishing priorities for PC-QI implementation [17]. Our aims were consistent with characteristics of an NGT process. We sought to identify areas of consensus and establish priorities for implementing the PC-QIs: individual prioritization, group discussion for the purpose of consensus building, and final prioritization of the PC-QIs and strategies [19]. The benefits to using a NGT approach include: it is resource efficient (requires less time and money), requires little preparation by participants (especially important for busy clinicians and volunteer Patient Partners), allows for in-session completion during face-to-face meeting, and encourages equal input from different perspectives [19, 23].

As part of our mixed methods design, both quantitative and qualitative data were collected across three ‘rounds’ of activities during the consensus process. This mixed methods approach served two purposes: 1. To use both quantitative and qualitative data to provide a more comprehensive view of our findings in the first round of activities and inform the second round of activities, and 2. To use qualitative data from the second round to inform the third round of data collection, where quantitative data was collected. Additional details regarding how qualitative and quantitative data were used to inform each subsequent round is described below under our presentation of the application of the NGT.

### Identification of the PC-QIs

The PC-QIs were developed by Santana et al. through a multi-phased program of research which involved a review of the literature [24, 25] and co-design with patients, caregivers, community members, healthcare providers, policymakers, and quality improvement experts in Alberta, across Canada, and internationally. Stakeholders were engaged through a survey, interviews, focus groups, and a modified Delphi consensus process [6, 26–28]. The PC-QIs were developed based on the Donabedian model for healthcare improvement (evaluates the structures, processes, and outcomes of care) [29]. The list of PC-QIs has been published [6] and was presented to the panelists for their review and prioritization for this study.

### Development of an initial list of implementation strategies

We identified implementation strategies through a rigorous research process using implementation science to assess Canadian healthcare organizations’ readiness to implement and use PC-QIs (via survey) and identify barriers and facilitators to implementation (via interviews), from the perspective of Canadian healthcare organizations and primary care stakeholders in Alberta. These findings will be published elsewhere.

The study team identified the implementation strategies from key aspects of organizations’ readiness, barriers, and facilitators that emerged from the previous study, considering: 1. the salience of the construct across stakeholder groups interviewed, based on our analysis using the Consolidated Framework for Implementation Research (CFIR) [30]; and 2. the number of codes for each construct. These factors were mapped to evidence and theory-informed broad strategies using the CFIR-ERIC (Expert Recommendations for Implementation Change [31]) Implementation Strategy Matching Tool. The broad strategies chosen included those that have been endorsed by the greatest proportion of experts to be among the “top seven” strategies to address a particular barrier. We grouped the broad strategies thematically and further developed the strategies to reflect the context of PC-QI implementation in primary care in Alberta. The interview data (transcripts) directly informed the specific wording used to formulate the strategies. A summary of the mapping process is accessible via [Additional file 1].

### Setting

The province of Alberta in Canada is home to over four million people and the country’s fourth most populous province. There are five health zones in Alberta (North, Edmonton, Central, Calgary and South) that serve diverse areas of the province including both rural and urban areas. While Alberta Health Services is considered the province’s single health authority, it has a limited role in the delivery of primary care, which is mainly provided by family physicians, paid by the provincial Ministry of Health (Alberta Health), on a fee-for-service basis [32]. Primary care clinics in Alberta vary in their size, resources available, and composition of staff. Most primary care clinics tend to be smaller practices, however, there are also larger “academic” clinics that provide training to medical interns and residents and conduct research. Most of the academic clinics are situated in the largest urban centres of Edmonton and Calgary. These clinics may also have dedicated staff for quality improvement.

Primary Care Networks (PCNs) in Alberta provide support to member family physicians (and their clinics), generally based on geography, with the vast majority of primary care physicians in Alberta being part of a PCN. Within PCNs there are also centralized, distributed and mixed models of operation. The centralized PCN model more closely replicates a referred outpatient program typical of the acute care system. Member physicians or nurse practitioners refer patients to the geographically distinct PCN central clinic for services. Distributed PCNs provide most resources within the community-based clinics that are the “medical home” for patients that are attached to a primary care provider, who is a PCN member. Mixed models can support logistical realities, such as lack of space within member clinics and “economy of scale” benefits of centralization.

### Panel recruitment

The consensus panel was selected to ensure a diverse representation of primary care stakeholders in Alberta. We targeted approximately 9–12 stakeholders to ensure geographical and role representation of stakeholder groups as well as considering efficient and constructive group processes [23]. We aimed to obtain representation from the five health zones in Alberta as well as perspectives from both urban and rural areas. Stakeholder groups targeted included: patients (2–3), physicians (2–3), clinic QI managers/leads of large academic clinics (2–3), and QI staff at PCNs (2–3).

We recruited potential consensus panelists from among past interview participants who provided their perspectives on barriers and facilitators to implementing the PC-QIs through email invitation. Patient partners were recruited through the Alberta Strategy for Patient-Oriented Research (SPOR) Patient Engagement Platform via a posting on their website, as well through email. We also approached the Alberta SPOR Primary Care and Integrated Health Care Innovation Network’s Patient and Families’ Panel for recruitment of interested patients. The invitation included an overview of the study aim and objectives, the consensus process, and time requirements for participation. Panelists were offered compensation for their participation with a $50 electronic gift card.

### Application of an adapted NGT

We used a modified approach to NGT. First, the steps of an NGT were broken down into three ‘rounds’ of activity to accommodate virtual participation during the COVID-19 pandemic and to minimize time burdens on panelists, including clinicians, staff working at and with primary care clinics, and volunteer Patient Partners. The rounds included: Round 1 - A remote round of prioritization (administered by email) of PC-QIs and strategies individually by panelists; Round 2 - A virtual “face-to-face” Zoom meeting of all panelists to discuss results from Round 1, where panelists provided additional insight into their prioritization; and Round 3 - A remote round of re-prioritization (administered by email) of PC-QIs and strategies individually by panelists, considering the discussion in Round 2. Another adaptation made was that the ‘silent generation of ideas’ component of an NGT [23] was replaced with a presentation of previous study results, where extensive engagement of stakeholders in PCC measurement and primary care in Alberta was done (as described above).

#### Round 1 process

We developed a survey/prioritization tool using ‘Qualtrics’ [33], a web-based data collection and management platform, supported by the University of Calgary. The study team pre-tested the survey for clarity and ease of use. The survey included two main sections, designed to take approximately one hour to complete:

1. A list of all 26 PC-QIs, with hyperlinks to their full descriptions (including definitions, technical specifications, relevant data sources, and supporting evidence), of which panelists were asked to prioritize their top five choices for implementation in primary care in Alberta. Panelists were also asked to include comments about their prioritization choices in a free-text box.
2. A list of ten strategies (including full descriptions) for implementing the PC-QIs in primary care in Alberta, which the panelists were asked to rank from most to least important. Using this ranking process, we sought to obtain a general sense of how the strategies were perceived by the panelists, which could be further discussed in Round 2, during the virtual meeting component. Panelists were asked to include any missing strategies or comments about their ranking choices in a free-text box. The strategy descriptions are presented in Table 1 below.

**Table 1:** Description of PC-QI implementation strategies (includes changes based on round 2)

Implementation strategy	Description
Needs assessment	Engage Primary Care Networks, clinics, and patients ***about their understanding of person-centred care*** and needs/***priorities*** around measuring person-centred care (considerations: obtain buy-in from stakeholders)
Develop partnerships	Coordinate potential partners who can collaborate on supporting implementation of the PC-QIs, including Universities, the Health Quality Council of Alberta, Primary Care Networks, patient advisories/groups, Alberta Health, and Alberta Health Services (considerations: Clinics require support from partners to enhance capacity for implementing successfully and sharing of resources)
Obtaining quality improvement resources	Engage leadership at Alberta Health and/or Alberta Health Services regarding resources for quality improvement in primary care, which may include: additional dedicated staff and/or physician compensation or funding models to support time spent on quality improvement, supporting electronic systems to help with data collection from patients (e.g. tablets), managing the data and making it accessible to providers (e.g. Electronic Medical Records), and dashboards that will show providers the results in a more timely way (considerations: would address resource constraints and competing priorities for physicians; clinics that have these electronic systems in place are better able to use the PC-QIs in a way that is easy for their staff)
Aligning measurement efforts	Hold meetings with key primary care stakeholders who are involved in guiding and mandating measurement in the province, including: Alberta Health, The College of Physicians and Surgeons of Alberta, and the Accelerating for Change Transformation Team under the Alberta Medical Association (considerations: improving patient experience is a provincial priority, avoid extra measurement burden on staff, helps with motivation to use the PC-QIs if mandated and tied to funding, helps with primary care staff and provider motivation, avoids duplication of efforts)
Support from partners for implementation	Coordination with the Health Quality Council of Alberta and Primary Care Networks who can support clinics with distributing patient surveys, collecting the data from the surveys, and reporting on the PC-QIs to the clinics, supporting the clinics to make improvements based on the data (considerations: minimize clinic staff time and resources and minimize conflicting priorities for focussing on patient care)
Champions	Identify and work with "champions" (those who actively promote person-centred care measurement, including physicians, primary care network staff, and patients) to engage clinic staff on person-centred care and the importance of measurement to improve patient experiences and outcomes (considerations: some healthcare providers do not see the value or are familiar with the research, may address motivation challenges)
Adapting patient surveys	Work with patients and primary care staff to tailor the surveys for patients to ensure they meet their needs of providers and patients (considerations: surveys have tended to be long for patients and providers/quality improvement staff do not see the value or do not feel they can make any improvements). Note: tailoring the surveys will require collaboration with researchers to make sure the questions have some scientific basis to ensure the information is "valid" (can be trusted)
Patient engagement	Working with primary care staff and patient groups to engage patients ***generally and at the clinic level*** around the value of completing patient experience surveys and the importance of their feedback for improving person-centred care (considerations: patients do not complete surveys, especially if they are long; ***engagement is needed with patients around expectations for care – higher expectations, better outcomes. Will get more valuable engagement. Providers will see this and help to shift practice)***
***Co-designing materials*** to implement the PC-QIs	***Co-design*** packaging of PC-QIs ***with providers, patients, and primary care organizations to provide tools*** that clearly show how to measure (what questions you need to ask on a survey), what the research shows to support measuring a particular indicator, and examples on what changes can be made to improve on an indicator (considerations: showing the value of why you would use an indicator, making it easy for those using the indicators to see what to do; ***provide “change packages” to help guide clinics on how to act on their data; while some standards can be established caution is needed around a “cookie cutter approach” – flexibility and tailoring is needed to accommodate specific clinic needs (e.g. rural vs. urban/smaller centres vs. larger); provides an opportunity to for patients and providers to work together)***
Education for clinical staff	Organize meetings with ***all*** clinic staff to orient them ***on person-centred care*** and using the PC-QIs, including how to collect the data from patients, summarize it using the PC-QIs and make changes to how care is delivered to improve on the indicators (considerations: not all staff know how to do quality improvement, dedicated staff usually needed, use of the PC-QIs would be led by the clinics vs. external partner)
**Pilot the implementation	Identify clinics that will be involved in modelling and simulating the implementation of the PC-QIs to learn how best to collect data, report, and feedback to clinics for quality improvement. Identifying an effective process for implementation and demonstrating it will empower champions to build momentum among other primary care practices (considerations: will show clinics that it is feasible and effective, allow clinics to try it out without long-term commitment)

Bold and italic font indicates changes made to strategies based on Round 2 discussions

**indicates a new strategy that was developed from Round 2

The rationale for prioritizing PC-QIs and strategies is based on recommendations which suggest focussing on 3–5 strategies is optimal [34].

### Materials

Along with the survey link, each participant received the following materials: 1. Background information about the study; 2. A summary of the findings from the survey conducted on organizational readiness to implement the PC-QIs and the interviews on barriers and facilitators to implementation; and 3. The full monograph of the PC-QIs, which included background information on their development and descriptions of each PC-QI. Panelists were asked to complete the survey within two weeks.

### Analysis

We analyzed the results from Round 1 of the consensus process using a mixed methods approach to enhance our interpretation of the findings [23]. To determine overall relative priority of the PC-QIs across panelists and stakeholder groups, we first calculated the frequency of each PC-QI as one of the top five priorities [35]. To determine the relative popularity of each PC-QIs as a top five priority, we calculated median ranking and interquartile range for each PC-QI that was prioritized, as well as the minimum and maximum range rating. Analysis was conducted using Microsoft Excel. To summarize the results for the panelists, we created a bar graph to show the relative popularity of each PC-QI that was indicated as a top five priority among all panelists. A colour-coded summary table was also developed to guide discussion (green = high relative priority, among top five across panelists; yellow = medium relative priority, among many of the panelists; red = low relative priority, only prioritized by zero, one, or two panelists). Any qualitative feedback (quotes) provided by panelists was also summarized and shared with the panelists for their consideration for Round 2. Specific feedback provided for individual PC-QIs and strategies were presented to panelists with the scores as a joint display to provide a more comprehensive summary of the results [36].

#### Round 2 process

A 1.5 h virtual meeting was conducted via zoom with all the panelists, including: welcome and introductions; consent to record the meeting; presentation of the PC-QI prioritization and strategy ranking results and questions from panelists; facilitated group discussion by one member of the study team regarding the response to findings (agreement/disagreement, issues to consider – priority for whom, feasibility, value added, and clear PC-QI and implementation strategy priorities. Notes were taken by another member of the study team.

### Materials

The meeting agenda, bar graphs, summary table, and panelist comments were shared with panelists one week prior to the consensus meeting for optional review. Reference materials were also provided, including the definitions for the PC-QIs and the strategies.

### Analysis

A verbatim transcript was produced from the Zoom meeting recording. We conducted a content analysis of the transcript and meetings notes to inform any potential changes to the prioritization of the PC-QIs and strategies [37]. A summary of the qualitative feedback and any changes made to the implementation strategies was developed.

#### Round 3 process and materials

As in Round 1, the study team developed and pre-tested the final survey using the Qualtrics platform. It included three sections, designed to take approximately 30 min to complete:

1. A summary of the PC-QI prioritization with notes (qualitative data) based on the Round 2 group discussions. Panelists were presented with the prioritized list of PC-QIs, including lower prioritized PC-QIs that were discussed in Round 2 (those lower prioritized and not discussed were discarded from consideration) and their scores.
2. A summary of the implementation strategy prioritization and scores, with notes (qualitative data) based on the Round 2 group discussions. Panelists were presented with the ranked list of strategies that had been revised or added to, based on Round 2 discussions.
3. A brief survey regarding their experience as a panelist (5-point Likert scale), covering topics such as: value of the consensus process for informing PC-QI implementation, structure of the consensus process, whether panelist input was valued, burden of the consensus process, and interest in further involvement in future PC-QI implementation.

Each panelist was sent their individual prioritization and ranking from Round 1 as a reference. Panelists were also asked to consider the discussion from Round 2 to re-prioritize the PC-QIs and strategies. Panelists were asked to complete the survey within two weeks.

### Analysis

The analysis was similar to Round 1 for the PC-QI prioritization. Analysis for the implementation strategy prioritization followed processes done for the PC-QI prioritization as well. A summary of any final changes to the prioritization as well as feedback was shared with panelists and with primary care stakeholders for feedback and review.

## Results

### Panel

A total of 11 panelists participated in the consensus process. A summary of the panel composition is found in Table 2 below.

**Table 2:** Summary table for consensus panelists

Panelists (N = 11)	Proportion % (n)
*Perspective*	
Patient	27.3% (3)
Primary care physician	27.3% (3)
Primary care network QI staff	27.3% (3)
QI staff (large primary care clinic)	18.2% (2)
*Gender*	
Woman	72.7% (8)
*Alberta zone representation*	
Provincial/patient partner*	27.3% (3)
North zone	9.1% (1)
Edmonton zone	18.2% (2)
Central zone	9.1% (1)
Calgary zone	27.3% (3)
South zone	9.1% (1)

*Patient panel members were not recruited to represent a specific zone, but rather to provide a general/provincial patient perspective

10/11 panelists participated in all three rounds of the consensus process, with one panelist unable to complete the first round of the consensus process due to work commitments. All health care delivery zones in Alberta were represented by panelists, including those that served both urban and rural geographic areas. Three panelists represented academic primary care clinics in larger urban areas with high levels of capacity in QI. One of these panelists also represented an urban academic clinic that serves a large immigrant and newcomer community.

### Person-centred quality indicators

**Fig. 1:**
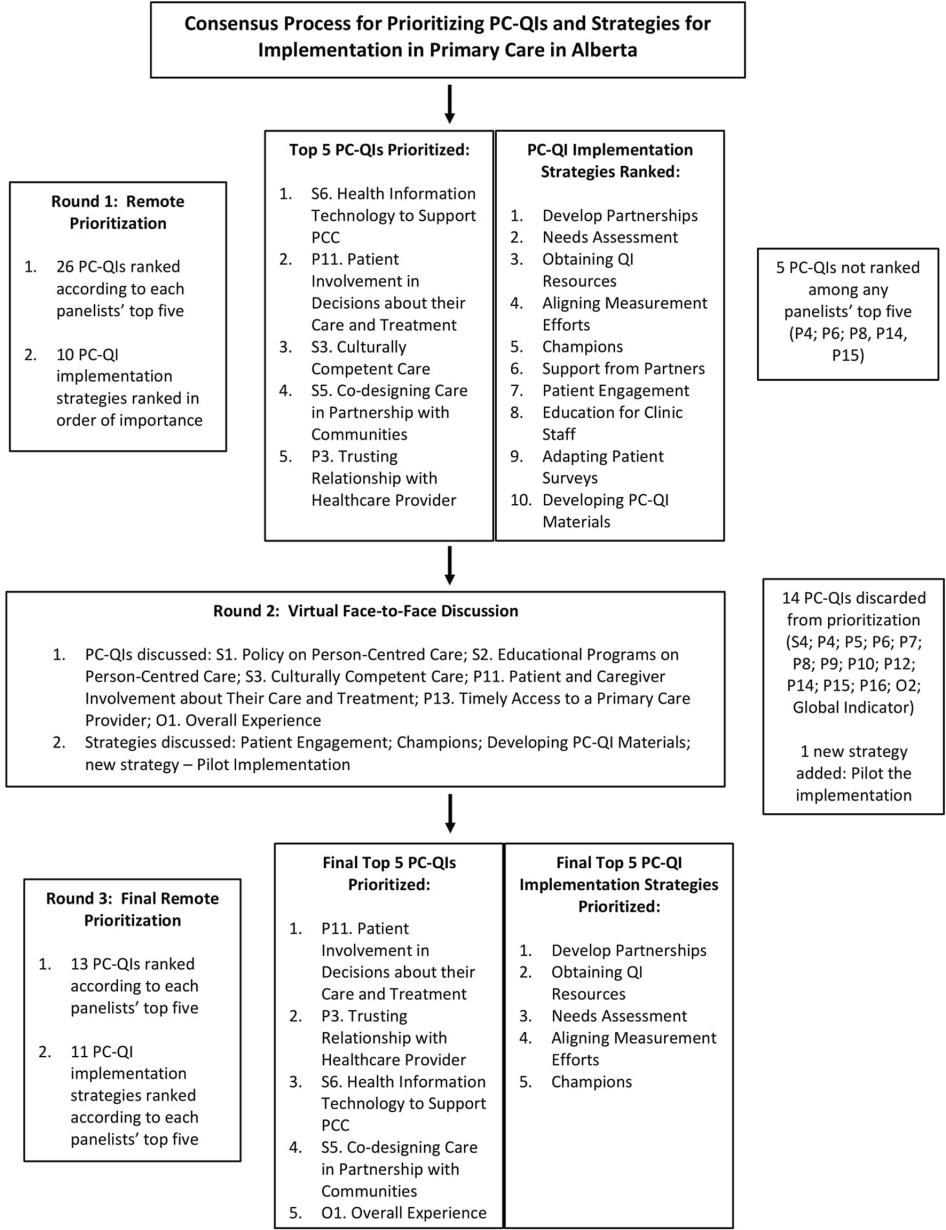
Consensus process for prioritization PC-QIs and strategies for implementation

The final PC-QIs prioritized by the panelists from the consensus process and the process of prioritization are presented on Fig. 1 below.

In the first round of individual remote prioritization by the panelists, the ‘PC-QI for Health information technology to report on PCC’ was highly prioritized across stakeholders, but not by patients. This highlights the importance of having structures necessary for using the PC-QIs, something important from an implementer perspective. On the other hand, the second prioritized PC-QI ‘Patient and caregiver involvement about decisions in care’, was highly prioritized by all three patient panelists. The remaining priorities for top five were prioritized across stakeholder groups, showing greater consensus of their relative importance.

During the Round 2 virtual meeting, in general, panelists agreed with the PC-QI prioritization. However, panelists discussed how PC-QIs that were prioritized may be more challenging to implement, compared to those seen as lower priorities (and in some cases already being measured). Concerns around implementation were mainly around getting clinics to collect this data (feasibility), see it as being able to be measured (relevance), feeding back the results to the clinics, and making changes to act on the data. Panelists noted that it is important to balance the likelihood that an indicator will support actual change (“actionability”) versus the feasibility of implementation, including resource requirements. Panelists also discussed PC-QIs that were not highly prioritized including: ‘Timely access to a primary care provider’; ‘Overall experience (patients’ overall rating of their care experience in a health facility)’; and ‘Policy on person-centred care’. While these PC-QIs were not highly prioritized by the group, individuals had ranked them among their top PC-QIs, resulting in some discussion on the discrepancies between rankings and differences in perspectives on relative importance.

Panelists reaffirmed the top five priority PC-QIs from Round 1 (though different rank order) in Round 3, except for the PC-QI for ‘Overall experience’ (previously low priority), which became one of the top five priorities across panelists. The PC-QI ‘Timely access to a primary care provider’ was also ranked higher in Round 3 – considered a “medium” priority across panelists. The results suggest relatively high-level consensus regarding PC-QI prioritization and that the Round 2 discussions did change some perspectives around the indicator prioritization. The details regarding PC-QI prioritization for all three rounds are summarized on Table 3 below.

**Table 3:** Summary of consensus results for PC-QI prioritization, by round

Person-centred quality indicator	Round 1: prioritization (N = 10)			Round 2: discussion (N = 11)	Round 3: prioritization (N = 11)		
	Rank (frequency in Top 5)	Median (IQR)	Stakeholder group priority		Rank (frequency in Top 5)	Median (IQR)	Stakeholder group priority
*Structure indicators*							
S1. Policy on person-centred care	13 (1)	1 (1–1)	1 PCN	Concern where policy is sometimes at odds with patient care and at times even PCC (what matters to the patient)	10 (2)	3 (2–4)	1 Patient; 1 PCN
				Policy as a necessary overarching structure that will enable are more shared understanding of PCC and goals for improvement			
				Importance of having both policy, which comes from the top, as well as a grassroots movement to see actual improvements			
S2. Educational programs on person-centred care	13 (1)	1 (1–1)	1 PCN	Important to bring the healthcare workforce on the same page in terms of understanding PCC and it is both actionable and measurable	12 (1)	2 (2–2)	1 PCN
				Education could fall under S7. Structures to report on PCC Performance			
S3. Culturally competent care	3 (4)	1.5 (1–2.25)	1 Physician; 2 Patient; 1 PCN	Concern about measuring this as it can be delicate and will mean different things to different people. It may be challenging to measure various aspects of culturally competent care in a way that is tangible and meaningful	7 (4)	3 (2.75–3.25)	1 Patient; 1 Physician; 2 PCN
S4. Providing an accommodating and supportive person-centred care environment	15 (1)	5 (5–5)	1 Patient	(Not discussed)	N/A	N/A	N/A
S5. Co-designing care in partnership with communities	4 (4)	2 (1.75–2.75)	1 Clinic QI Staff; 2 Patient; 1 Physician	(Not discussed)	4 (7)	4 (4–4)	2 Patient; 3 Physician; 2 QI Staff
S6. Health information technology to support person-centred care	1 (5)	3 (1–4.25)	2 Clinic QI Staff; 2 PCN, 1 Physician	(Not discussed)	3 (7)	3 (1–3.5)	3 Patient; 2 Physician; 2 PCN; 1 QI Staff
S7. Structures to report person-centred care performance	9 (3)	4 (4.5–5)	1 Clinic QI Staff; 1 PCN; 1 Physician	(Not discussed)	13 (1)	5 (5–5)	1 PCN
*Process indicators*							
P1. Compassionate care	7 (3)	3 (2.5–3)	1 Patient;1 PCN, 1 Physician	(Not discussed)	9 (2)	1.5 (1.25–1.75)	1 Patient; 1 QI Staff
P2. Equitable treatment	6 (3)	2 (2–3)	2 Patient; 1 Physician	(Not discussed)	8 (4)	4.5 (3.5–5)	2 Patient; 1 Physician; 2 PCN
P3. Trusting relationship with healthcare provider	5 (4)	2.5 (1–4.25)	1 Clinic QI Staff; 2 Patient; 1 Physician	(Not discussed)	2 (7)	1 (1–2.5)	2 Patient; 2 Physician; 2 PCN; 1 QI Staff
P4. Accessing interpreter services	N/A (0)	N/A	N/A	(Not discussed)	N/A	N/A	N/A
P5. Communication with healthcare system	11 (2)	3.5 (3.25–3.75)	2 Clinic QI Staff	(Not discussed)	N/A	N/A	N/A
P6. Communication between patient and healthcare provider – nurse	N/A (0)	N/A	N/A	(Not discussed)	N/A	N/A	N/A
P7. Communication between patient and healthcare provider – physician	14 (1)	2 (2–2)	1 Patient	(Not discussed)	N/A	N/A	N/A
P8. Information about taking medication	N/A (0)	N/A	N/A	(Not discussed)	N/A	N/A	N/A
P9. Communicating test results	13 (1)	1 (1–1)	1 PCN	(Not discussed)	N/A	N/A	N/A
P10. Coordination of care	8 (3)	4 (4–4.5)	1 Patient; 1 PCN, 1 Physician	(Not discussed)	N/A	N/A	N/A
P11. Patient and caregiver involvement in decisions about their care and treatment	2 (5)	3 (3–4)	3 Patient; 2 PCN	Important for patients to work with their family physician on care and treatment as there is a long-term relationship. This can be considered as an umbrella indicator that would include things like compassionate care, communication, etc	1 (10)	3 (2–4.5)	3 Patient; 3 Physician, 3 PCN; 1 QI Staff
P12. Engaging patients in managing their own health	15 (1)	5 (5–5)	1 PCN	(Not discussed)	N/A	N/A	N/A
P13. Timely access to a primary care provider	10 (2)	2 (1.5–2.5)	1 Clinic QI Staff; 1 PCN	Surprise about the low prioritization as this may be important to patients	6 (4)	2.5 (1.75–3.5	1 Patient; 1 Physician; 2 QI Staff
				Patients tend to be happy with their care if they have access/they have a doctor (so experience results can be biased)			
				Panelists shared experiences with this indicator not actually resulting in real improvements/changes			
P14. Patient preparation for a planned treatment program	N/A (0)	N/A	N/A	(Not discussed)	N/A	N/A	N/A
P15. Transition planning	N/A (0)	N/A	N/A	(Not discussed)	N/A	N/A	N/A
P16. Using patient-reported outcomes to deliver patient-centred care	12 (2)	2 (1.5–2.5)	1 Clinic QI Staff; 1 PCN	(Not discussed)	N/A	N/A	N/A
*Outcome indicators*							
O1. Overall experience	13 (1)	1 (1–1)	1 Clinic QI Staff	It would be important to be able to evaluate a patient’s whole journey as they interact with various aspects of the healthcare system (not just communication with nurse or physician)	5 (5)	3 (3–4)	1 Patient; 1 Physician; 2 PCN; 1 QI Staff
O2. Cost of care - affordability	15 (1)	5 (5–5)	1 PCN	(Not discussed)	N/A	N/A	N/A
Global indicator: friends and family test	13 (1)	1 (1–1)	1 Physician	(Not discussed)	N/A	N/A	N/A

Person-Centred Quality Indicators (PC-QIs) are referenced according to their categorization as being a “structure” (‘S’), “process” (‘P’), or “outcome” (‘O) indicator for evaluating the quality of care [22]. PCC refers to person-centred care; QI refers to quality improvement; PCN refers to Primary Care Network; IQR refers to the interquartile range

### Implementation strategies

The final strategies prioritized by panelists are included on Fig. 1.

In Round 1, there was less consensus around strategies, which may indicate the difficulty in being able to rank. Panelist comments indicated that some panelists viewed all or most strategies important but attempted to rank based on an “order” in which the strategies could be implemented in practice rather than their potential impact. While this was not intended (instructions indicated ranking based on importance/potential impact), this approach was discussed in Round 2.

Discussion in Round 2 regarding the implementation strategies highlighted an overall concern regarding the feasibility of implementing the PC-QIs. To some panelists, feasibility was most important (and thus, informed their ranking). Panelists discussed how a system-level approach to implementation would be challenging given the resource constrained system and the varied contexts for primary care implementation (PCN or clinic capacity, interest, and budgetary constraints).

Panelists also discussed the importance of co-design among stakeholders to plan for implementation and the potential value of piloting PC-QI implementation to demonstrate the effectiveness and value using the PC-QIs to improve the quality of care. This resulted in modifications to the potential implementation strategies.

For Round 3, among the strategies, those that were most highly ranked in Round 1 were re-affirmed among the top five strategies. The order changed somewhat, but ‘Developing partnerships’ was confirmed as the top strategy for system level implementation. A notable change in ranking was for ‘Co-designing materials for PC-QI implementation’, which was prioritized higher during this round, based on some of the discussions in Round 2 (changes to reflect more emphasis on co-design). The details regarding strategy prioritization for all three rounds are summarized on Table 4 below.

**Table 4:** Summary of consensus results for strategy prioritization, by round

PC-QI implementation strategy	Round 1: prioritization (N = 10)		Round 2: discussion (N = 11)	Round 3: prioritization (N = 10)		
	Rank	Median (IQR)		Rank (frequency in Top 5)	Median (IQR)	Stakeholder group priority
Needs assessment	2	2 (1.25–3.75)	There is a need for general engagement and assessment of primary care stakeholders for buy-in and ensuring everyone is on the same page in terms of what is meant by PCC (although it was recognized that the PC-QIs are intended to help define)	3 (6)	2 (2–2)	2 Patient; 1 Physician; 3 PCN
Develop partnerships	1	2 (1–4.75)	There isn’t a need to create new partnerships	1 (8)	1 (1–2)	3 Patient; 2 Physician; 1 PCN; 2 QI Staff
Obtaining quality improvement resources	3	4.5 (3.25–5)	(Not discussed)	2 (7)	3 (3–4.5)	2 Patient; 2 Physician; 2 PCN; 1 QI Staff
Aligning measurement efforts	4	5.5 (4.25–7.5)	Greater alignment will come from engagement of both providers and patients	4 (6)	3.5 (3–4)	2 Physician; 3 PCN; 1 QI Staff
Support from partners for implementation	6	6 (5–7)	(Not discussed)	10 (1)	4 (4–4)	1 Physician
Champions	5	6 (3–6.75)	Champions are needed to spearhead implementation efforts, but require the data to demonstrate effectiveness	5 (6)	4.5 (2.5–5)	2 Patient; 1 Physician; 2 PCN; 1 QI Staff
Adapting patient surveys	9	7.5 (7–8.75)	(Not discussed)	N/A (0)	N/A	N/A
Patient engagement	7	7 (3.25–8.75)	Surprise about patient engagement being ranked so low, although patient engagement was seen to fit under “develop partnerships”	7 (4)	2.5 (2–3)	2 Patient; 1 PCN; 1 QI Staff
			Patient engagement was seen as being critical and needed from the start as part of a co-design process for implementation and to begin changing the norms of providers			
			Some distinction was also made around the need for clinic level engagement and wider engagement as it relates to patient expectations around their care			
Co-designing materials to implement the PC-QIs	10	8.5 (5.5–9.75)	Surprise around the low ranking of “developing PC-QI materials”	8 (3)	3 (2–4)	1 Patient; 1 Physician; 1 PCN
			Tensions between having standards around tools, but also having some flexibility to tailor based on clinics’ needs			
			Suggested co-designing materials with stakeholders to optimize design of materials			
Education for clinical staff	8	7.5 (4.75–8.75)	(Discussion partly captured under Needs Assessment)	6 (5)	5 (3–5)	2 Patient; 1 PCN; 2 QI Staff
**Pilot the Implementation	N/A	N/A	Piloting the implementation where it can be demonstrated that the data can be collected, fed back, and changes made would support implementation. Adjustments to implementation can be made (e.g. after 3, 6, 9 months) based on learning from the pilot	9 (2)	4 (4–4)	1 Patient; 1 PCN

Panelists asked to rank all ten strategies in round 1; prioritization in top five not used to determine ranking

**indicates a new strategy that was developed from Round 2 (not ranked in round 1). PC-QIs refer to Person-Centred Quality Indicators; PCC refers to person-centred care; QI refers to quality improvement; PCN refers to Primary Care Network; IQR refers to the interquartile range

### Panel evaluation

In general, panelists considered the consensus process to be positive and indicated future interest in being engaged in implementation. Table 5 provides a summary of the panelist responses related to evaluating the consensus process.

**Table 5:** Panelist evaluation of consensus process

Consensus process indicator	Agree	Neither	Disagree
Consensus process is an important step for informing PC-QI implementation	100% (9/9)	0% (0/9)	0% (0/9)
The structure of the consensus process was useful for obtaining input and group discussion	100% (10/10)	0% (0/10)	0% (0/10)
As a panelist, I felt my input was valued	80% (8/10)	20% (2/10)	0% (0/10)
I felt the activities were burdensome in terms of time and energy required for participation	11% (1/9)	11% (1/9)	78% (7/9)

“Agree” includes responses for “Strongly Agree” and “Agree,” “Neither” relates to the response “Neither Agree Nor Disagree,” and “Disagree” includes responses for “Disagree “or “Strongly Disagree.”

7 of 9 (78%) panelists that responded to the section of the survey on future involvement indicated an interest in working with stakeholders (government, primary care providers, patients, partners) to implement the PC-QIs in Alberta.

## Discussion

We aimed to contribute to the development of practical guidance on implementing the PC-QIs to promote adoption and use of the PC-QIs in primary care in Alberta, Canada. As a first step in this process, we undertook a consensus process to prioritize the PC-QIs and strategies for implementation. Consensus panelists, representing diverse perspectives in primary care in Alberta prioritized the following five PC-QIs for implementation in primary care in Alberta in the following order: ‘Patient and caregiver involvement in decisions about their care and treatment’; ‘Trusting relationship with healthcare provider’; ‘Health information technology to support PCC’; ‘Co-designing care in partnership with communities’; and ‘Overall experience’. Five evidence and theory-based PC-QI implementation strategies were also prioritized in the following order: ‘Develop partnerships’; ‘Obtain QI resources’; ‘Needs assessment’; ‘Aligning measurement efforts’; and ‘Champions’. With respect to priorities, those among the top five for both the PC-QIs and the implementation strategies did not change substantially between Rounds 1–3. However, the discussion in Round 2 did result in greater consensus as the PC-QIs that were prioritized were considered top priorities across stakeholder groups, rather than having specific PC-QIs that were more important to those implementing versus patients, for example. This indicates that the discussion was important for understanding different perspectives and consensus building.

Panelists indicated that there was value in the consensus process, where all panelists who completed the evaluation saw the process as an important step for informing PC-QI implementation and that it was useful for obtaining input from panelists and facilitating group discussion. Moreover, almost all panelists felt that their input was valued and that the activities were not burdensome in terms of time and energy for prioritization. This suggests potential for integrating the theoretical and evidence-based approaches of implementation science – presenting panelists with implementation strategies already informed by rigorous and wide stakeholder engagement and identified through a systematic process - into pragmatic, well-established participatory methods, such as the NGT. Moreover, our adapted NGT approach enabled the participation of diverse stakeholders in primary care by offering a modified process for input that accommodated the busy schedules of primary care providers, valued the time of volunteers, and addressed challenges associated with conducting participatory research in the context of COVID-19, where in-person discussion has been limited. The benefits of modifying the NGT process to facilitate greater participation of stakeholders for healthcare implementation science projects is also documented in work done by Rankin et al. [38]. Additionally, previous experiences with the NGT process have shown that valuing participant perspectives equally results in patient involvement being able to shift quality of care priorities among the group [39]. As such, for our study, we have “weighted” all panelist perspectives equally.

We were interested to note the relatively low prioritization of the PC-QIs related to ‘Timely access to a primary care provider’, as this indicator is one that is already integrated within Patient-Reported Experience Measures (PREMs) used in primary care in Alberta (thereby highly feasible to implement), and where access to care is considered a key value of primary care [2]. However, panelists also discussed the possibility that patients may have positive experiences with their primary care provider (due to relationship, grateful to have a provider), despite challenges with access. Additionally, as a strategy ‘Patient engagement’ was not a top priority, despite stakeholder interest in PCC. It can be noted, however, that some panelists considered ‘Patient engagement’ as part of a broader strategy to ‘Develop partnerships’ with various stakeholders in primary care.

The panelists’ discussion in Round 2 provided greater insight into the complexity of issues associated with PC-QI implementation at a system-level in primary care in Alberta: a need to balance the potential impact on improving PCC, feasibility (investments required for resources and coordination), health provider readiness, and what stakeholders value in both the care they provide, as well as the care they receive. While the strategies were already identified using extensive stakeholder engagement, through the consensus process, the panelists provided valuable input to further refine the implementation strategies to reflect these context-specific considerations for primary care in Alberta. In most cases, under a fee-for-service model of physician remuneration, the resources and time that can be allocated for QI in primary care represents a short-term opportunity cost that may directly and negatively impact health services and patient care. Only eight percent of all Alberta health care spending was allocated to community-based, office-delivered primary care in 2018–19 [40]. While the potential value of using PC-QIs are well demonstrated, the clinician and (physician) community clinic owner-operator usually choose direct patient care over value-added processes that promise future benefit. This is why implementation of tools, such as PC-QIs are so critical in the resource scarce primary care system. Evidence suggests that investments to strengthen primary care can result in improved population health outcomes as well as decreased costs to the health system [5, 41]. Indeed, in an evaluation conducted by the Health Quality Council of Alberta in 2019, they found that two primary care clinics in Alberta that operate under alternative funding models (where providers are paid a prospective amount to cover services provided to patients within a specific period of time) not only demonstrated improvements in PCC, but also led to health system savings of $4.3 and $7.2 million (by each clinic) over the 2016–2017 period [42].

Our consensus process experience highlights the critical importance of highly participatory and continued engagement of stakeholders throughout planning for implementation and beyond in order to attain greater relevance and promote effective implementation that will translate into meaningful change [43–46]. This is consistent with perspectives that suggest that tailoring and adapting of interventions is best facilitated through participatory methods that ensure that those who will be involved in implementation and impacted by it are “at the heart” of planning for implementation [13, 47–50]. Moreover, the benefits of highly participatory approaches are not limited to specific projects, but can help to establish ongoing, collaborative relationships between researchers and stakeholders that synergize as partners build trust and continue to work together long-term towards healthcare system improvements [45, 47]. This is also reflected in our experience, where most of the panelists indicated an interest in continuing to collaborate and work with other stakeholders to implement the PC-QIs in Alberta. Many of these strengths or benefits associated with collaborative planning with diverse stakeholders have also been described outside the health care context as “social lab” methodology, used for addressing complex social system problems [49, 50].

A strength of our study is demonstrating how established participatory methods, such as consensus processes can support the systematic, theory and evidence-informed approach of implementation science. Our experience contributes to the ongoing reflection and dialogue regarding the development of approaches and methods for adapting and tailoring interventions to specific contexts. Furthermore, we were able to attain diverse representation on our consensus panel, representing healthcare providers, patients, QI staff, and PCN staff, from across Alberta, who could provide different perspectives on planning for PC-QI implementation. Finally, our mixed methods study design enabled us to use both quantitative measures (i.e. calculating ranking tendency measures) as well as qualitative feedback (i.e. comment boxes and discussions) to inform the prioritization of the PC-QIs and strategies, and to enhance our process for consensus building.

Limitations of our study include the relatively small representation of panelists for each stakeholder group, which may limit the generalizability of our results. While we strived to attain geographic and role diversity, we did not seek to ensure that the panelists represented other aspects of identity (e.g. cultural, socio-economic). The inclusion of additional stakeholders in primary care in Alberta who represent other aspects of diversity may influence the priorities identified. While there is no consensus on the ideal number of panelists, we opted to balance representation with the need to ensure efficient and constructive group processes [23]. Our future stakeholder engagement will involve validating our findings and obtaining additional input and feedback, which will also inform our planning for future implementation efforts. As well, it is important to note that while the modified approach to NGT enabled us to attain high levels of participation among the consensus panelists, there was insufficient time to discuss each PC-QI and strategy during the virtual meeting, which may have influenced our final results. This may have contributed to little change seen between prioritization of the PC-QIs and strategies between Rounds 1 and 3. However, we believe that the panelists chose to prioritize the discussions most important to them, and that the opportunities to provide additional qualitative feedback during the remote rounds and following the virtual meeting enabled participants to share any additional reflections with the panel for their consideration. It is also important to note that while presenting panellists with implementation strategies already informed by rigorous and extensive stakeholder engagement can be valuable, it may also be limiting and restrictive with regards to innovative thinking. Despite this potential limitation, we did find that panelists provided feedback to revise the strategies and propose a new one as well. Additionally, while NGT is an established consensus method, our specific adaptions to the NGT have not been tested previously. However, given the dearth of tools and documentation regarding the use of participatory approaches in planning for implementation and adapting to the local context [47], we believe that our experience contributes to the literature in this area. Finally, given that the evaluation of the process by the panelists was not anonymized, there is a risk that the responses were biased towards a positive experience.

Future research will incorporate ongoing stakeholder engagement, including many of our consensus panelists, to plan for implementation of the PC-QIs in Alberta. This will include meetings with provincial policymakers (Alberta Health), implementation partners (such as the Health Quality Council of Alberta and Alberta Health Services), PCNs, patients, and providers, to share our research findings, obtain additional input, and operationalize a provincial implementation strategy. This will include discussion on identifying appropriate measures for the PC-QIs. Additionally, while some panelists suggested that the implementation strategies may follow a specific sequence to ensure greater feasibility and impact, this will be discussed with the larger group of provincial stakeholders.

## Conclusions

In conclusion, our study demonstrates the value of co-designing and integrating participatory approaches into implementation science. Participatory approaches that engage diverse stakeholders in adapting PC-QI implementation to the context are highly valuable for obtaining buy-in for ongoing engagement, and ensuring the implementation is responsive to the local needs and will lead to meaningful changes the quality of care for patients.

## Supplementary Information

**Additional file 1.** The table provided shows where each implementation strategy is mapped to one or more broad strategy from the Consolidated Framework for Implementation Research (CFIR) - Expert Recommendations for Implementing Change (ERIC) Strategy Matching Tool.

## Data Availability

The datasets used and/or analysed during the current study are available from the corresponding author on reasonable request.

## Abbreviations

PCC: Person-centred care
PC-QIs: Person-centred quality indicators
QI: Quality improvement
NGT: Nominal group technique
PREMs: Patient-reported experience measures
PCNs: Primary care networks
CFIR: Consolidated framework for implementation research
