# Supplementary material for: Co-designing person-centred quality indicator implementation for primary care in Alberta: a consensus study
Source: Res Involv Engagem. 2022 Nov 8;8:59. doi: 10.1186/s40900-022-00397-z (PMC9641306; doi:10.1186/s40900-022-00397-z)
Supplement: Supplementary file 1 — Additional file 1. The table provided shows where each implementation strategy is mapped to one or more broad strategy from the Consolidated Framework for Implementation Research (CFIR) - Expert Recommendations for Implementing Change (ERIC) Strategy Matching Tool. [file 40900_2022_397_MOESM1_ESM.docx]

**Description of PC-QI Implementation Strategies (includes changes based on Round 2)**

| **Broad Strategies Identified from CFIR-ERIC Strategy Matching Tool**  (based on barriers and facilitators mapped to CFIR constructs) | **PC-QI Implementation Strategy** | **Description** |
| --- | --- | --- |
| Conduct a local needs assessment | Needs assessment | Engage Primary Care Networks, clinics, and patients ***about their understanding of person-centred care*** and needs/***priorities*** around measuring person-centred care (considerations: obtain buy-in from stakeholders). |
| Build a coalition, develop academic partnerships | Develop Partnerships | Coordinate potential partners who can collaborate on supporting implementation of the PC-QIs, including Universities, the Health Quality Council of Alberta, Primary Care Networks, patient advisories/groups, Alberta Health, and Alberta Health Services (considerations: Clinics require support from partners to enhance capacity for implementing successfully and sharing of resources). |
| Access new funding | Obtaining Quality Improvement Resources | Engage leadership at Alberta Health and/or Alberta Health Services regarding resources for quality improvement in primary care, which may include: additional dedicated staff and/or physician compensation or funding models to support time spent on quality improvement, supporting electronic systems to help with data collection from patients (e.g. tablets), managing the data and making it accessible to providers (e.g. Electronic Medical Records), and dashboards that will show providers the results in a more timely way (considerations: would address resource constraints and competing priorities for physicians; clinics that have these electronic systems in place are better able to use the PC-QIs in a way that is easy for their staff). |
| Alter incentive/allowance structures | Aligning Measurement Efforts | Hold meetings with key primary care stakeholders who are involved in guiding and mandating measurement in the province, including: Alberta Health, The College of Physicians and Surgeons of Alberta, and the Accelerating for Change Transformation Team under the Alberta Medical Association (considerations: improving patient experience is a provincial priority, avoid extra measurement burden on staff, helps with motivation to use the PC-QIs if mandated and tied to funding, helps with primary care staff and provider motivation, avoids duplication of efforts). |
| Facilitation, Promote network weaving, Organize clinician implementation meetings | Support from Partners for Implementation | Coordination with the Health Quality Council of Alberta and Primary Care Networks who can support clinics with distributing patient surveys, collecting the data from the surveys and reporting on the PC-QIs to the clinics, supporting the clinics to make improvements based on the data (considerations: minimize clinic staff time and resources and minimize conflicting priorities for focussing on patient care). |
| Identify and prepare champions | Champions | Identify and work with "champions" (those who actively promote person-centred care measurement, including physicians, primary care network staff, and patients) to engage clinic staff on person-centred care and the importance of measurement to improve patient experiences and outcomes (considerations: some healthcare providers do not see the value or are familiar with the research, may address motivation challenges). |
| Promote adaptability, Obtain and use patients/consumers and family feedback, Intervene with patients/consumers to enhance uptake & adherence, Develop academic partnerships | Adapting Patient Surveys | Work with patients and primary care staff to tailor the surveys for patients to ensure they meet their needs of providers and patients (considerations: surveys have tended to be long for patients and providers/quality improvement staff do not see the value or do not feel they can make any improvements). Note: tailoring the surveys will require collaboration with researchers to make sure the questions have some scientific basis to ensure the information is "valid" (can be trusted). |
| Prepare patients/consumers to be active participants, Involve patients/consumers and family members | Patient Engagement | Working with primary care staff and patient groups to engage patients ***generally and at the clinic level*** around the value of completing patient experience surveys and the importance of their feedback for improving person-centred care (considerations: patients do not complete surveys, especially if they are long; ***engagement is needed with patients around expectations for care – higher expectations, better outcomes. Will get more valuable engagement. Providers will see this and help to shift practice).*** |
| Develop educational materials, Develop a formal implementation blueprint | ***Co-designing Materials*** to Implement the PC-QIs | ***Co-design*** packaging of PC-QIs ***with providers, patients, and primary care organizations to provide tools*** that clearly show how to measure (what questions you need to ask on a survey), what the research shows to support measuring a particular indicator, and examples on what changes can be made to improve on an indicator (considerations: showing the value of why you would use an indicator, making it easy for those using the indicators to see what to do; ***provide “change packages” to help guide clinics on how to act on their data; while some standards can be established caution is needed around a “cookie cutter approach” – flexibility and tailoring is needed to accommodate specific clinic needs (e.g. rural vs. urban/smaller centres vs. larger); provides an opportunity to for patients and providers to work together).*** |
| Conduct educational meetings, Distribute educational materials | Education for Clinical Staff | Organize meetings with ***all*** clinic staff to orient them ***on person-centred care*** and using the PC-QIs, including how to collect the data from patients, summarize it using the PC-QIs and make changes to how care is delivered to improve on the indicators (considerations: not all staff know how to do quality improvement, dedicated staff usually needed, use of the PC-QIs would be led by the clinics vs. external partner). |
| Model and simulate change, stage implementation scale-up, capture and share local knowledge | **Pilot the Implementation | Identify clinics that will be involved in modelling and simulating the implementation of the PC-QIs to learn how best to collect data, report, and feedback to clinics for quality improvement. Identifying an effective process for implementation and demonstrating it will empower champions to build momentum among other primary care practices (considerations: will show clinics that it is feasible and effective, allow clinics to try it out without long-term commitment) |

*Note: Bold and italic font indicates changes made to strategies based on Round 2 discussions. **indicates a new strategy that was developed from Round 2.*
